# Genome-Wide Screening of Genes Required for Glycosylphosphatidylinositol Biosynthesis

**DOI:** 10.1371/journal.pone.0138553

**Published:** 2015-09-18

**Authors:** Yao Rong, Shota Nakamura, Tetsuya Hirata, Daisuke Motooka, Yi-Shi Liu, Zeng-An He, Xiao-Dong Gao, Yusuke Maeda, Taroh Kinoshita, Morihisa Fujita

**Affiliations:** 1 Key Laboratory of Carbohydrate Chemistry and Biotechnology, Ministry of Education, School of Biotechnology, Jiangnan University, Wuxi, Jiangsu, 214122, China; 2 Research Institute for Microbial Diseases, Osaka University, Suita, Osaka, 565–0871, Japan; 3 WPI Immunology Frontier Research Center, Osaka University, Suita, Osaka, 565–0871, Japan; Ruhr University Bochum, GERMANY

## Abstract

Glycosylphosphatidylinositol (GPI) is synthesized and transferred to proteins in the endoplasmic reticulum (ER). GPI-anchored proteins are then transported from the ER to the plasma membrane through the Golgi apparatus. To date, at least 17 steps have been identified to be required for the GPI biosynthetic pathway. Here, we aimed to establish a comprehensive screening method to identify genes involved in GPI biosynthesis using mammalian haploid screens. Human haploid cells were mutagenized by the integration of gene trap vectors into the genome. Mutagenized cells were then treated with a bacterial pore-forming toxin, aerolysin, which binds to GPI-anchored proteins for targeting to the cell membrane. Cells that showed low surface expression of CD59, a GPI-anchored protein, were further enriched for. Gene trap insertion sites in the non-selected population and in the enriched population were determined by deep sequencing. This screening enriched 23 gene regions among the 26 known GPI biosynthetic genes, which when mutated are expected to decrease the surface expression of GPI-anchored proteins. Our results indicate that the forward genetic approach using haploid cells is a useful and powerful technique to identify factors involved in phenotypes of interest.

## Introduction

Anchoring of cell surface proteins by the glycolipid glycosylphosphatidylinositol (GPI) is a conserved posttranslational modification in eukaryotes [1, 2]. Indeed, more than 60 proteins in *Saccharomyces cerevisiae* and more than 100 proteins in mammals are known to be modified with GPI. Moreover, GPI biosynthesis is essential for yeast growth and embryogenesis in mammals [3, 4]. It is synthesized by the stepwise addition of sugars, an acyl-chain, and phospho-ethanolamines to phosphatidylinositol (PI) in the endoplasmic reticulum (ER). GPI-anchored proteins (GPI-APs) are then remodeled and transported to the plasma membrane through the Golgi apparatus [5–7]. At least 17 steps are required for the correct biogenesis of GPI-APs in mammalian cells, including GPI biosynthesis, attachment to proteins, remodeling, and transport (S1 Table), and more than 25 genes directly involved in GPI biosynthesis have been identified.

Mutations in GPI biosynthetic genes result in several disorders. Somatic mutations in the phosphatidylinositol glycan class A gene (*PIGA*) in hematopoietic stem cells are causative of paroxysmal nocturnal hemoglobinuria (PNH), which is an acquired GPI deficiency [8]. PNH has also been shown to be caused by a combination of heterozygous germline mutations and somatic mutations in *PIGT* [9]. Recent progress in exome sequencing by the next generation sequencer (NGS) technology found that mutations in at least 12 GPI genes cause inherited GPI deficiencies (S1 Table) [10, 11], and the identification of genes required for GPI biosynthesis greatly contributes to our understanding of disease phenotypes and diagnosis.

To identify genes or proteins required for GPI biosynthesis, three major strategies have been used to date: yeast genetic screens, genetic screens using mammalian cells, and biochemical approaches (S1 Table). One of the advantages of using yeasts is that they stably maintain haploid states, which enables both forward and reverse genetics to be performed. Thus, genes involved in GPI biosynthesis have been identified using yeast mutants defective in the incorporation of tritiated inositol on proteins or α-agglutinin on the cell wall [4, 12]. However, the limitation of isolating mutant yeast cells is that most GPI biosynthetic genes are essential for growth, and their mutations cause severe phenotypes [6]. Biochemical approaches use co-immunoprecipitation with proteins that form functional complexes for biosynthetic reactions, and have identified subunits of the GPI-*N*-acetylglucosamine transferase (GPI-GnT) complex, dolichol-phosphate mannose (Dol-P-Man) synthase complex and GPI transamidase complex [13–15]. However, because most GPI biosynthetic proteins are multi-spanning transmembrane proteins, they can be difficult to isolate.

Genetic analysis using mammalian cells is an alternative method used to identify genes required for GPI biosynthesis [16] (S1 Table). It is based on the utilization or isolation of mutant cells defective in GPI-APs on the cell surface, and expression cloning of the genes responsible. Mutagenized mammalian cells such as T-lymphoma cells or Chinese hamster ovary (CHO) cells are often used, and 17 GPI biosynthetic genes have thus far been determined by expression cloning. A combination of mutant cell isolation and expression cloning involving mammalian cells can be a useful approach, but has several disadvantages. Clonal mutant cells have to be isolated, then the responsible genes from a cDNA library need to be determined from each mutant cell line.

Recently, several mammalian haploid cell lines have been reported [17–20]. Human HAP1 cells are one such adherent cell line derived from haploid KBM7 cells, and which can stably maintain the haploid state with one of each chromosome from 1 to 22 and the X chromosome [18]. Brummelkamp et al. previously used these cells to conduct genetic screening methods combined with gene trapping [18, 21], which is a method of disrupting genes by inserting trap vectors into an intron of an expressed gene to inhibit transcription and splicing. By combining haploid cells with gene trap methods, mutant cells can be obtained for phenotypes of interest, and the gene responsible for the mutant cells can be determined by sequencing the trapped site of the genome. In the present study, we used a forward genetic method involving haploid human cells and a combination of gene trap methods and NGS technology to identify genes required for GPI biosynthesis. Our results indicate that this genetic screening method is a powerful tool to help comprehend the GPI biosynthetic pathway.

## Materials and Methods

### Cells, Antibodies, and Reagents

HAP1 cells kindly provided by Thjin R. Brummelkamp (Netherlands Cancer Institute, Amsterdam, The Netherlands) were cultured in Iscove’s modified Dulbecco’s medium containing 10% fetal calf serum with necessary selection antibiotics [18]. Mouse monoclonal anti-CD59 (clone 5H8) and anti-DAF (clone IA10) were used as primary antibodies [22]. Phycoerythrin (PE)-conjugated goat anti-mouse IgG (Biolegend) was used as a secondary antibody. The bacterial pore-forming toxin proaerolysin and its variant fluorescent-labeled aerolysin (FLAER) were obtained from Protox Biotech [23].

### Plasmids

A retroviral gene trap vector, pCMT-SApA-BSD, containing an adenoviral splice acceptor (SA) site, a phosphoglycerate kinase 1 (PGK) polyadenylation (polyA) signal, a PGK promoter (PGKpro), the blasticidin-S deaminase (BSD) gene, and human growth hormone (hGH) polyA was constructed from pCMT-SAhygpA-NP21 [24], which was provided by Dr. Kyoji Horie (Nara Medical University, Nara, Japan). pCMT-SA was generated by amplifying the SA site using the primers GTTCCTATTCTCTAGAAAGTATAGGAACTTCAGTG (underline: *Xba*I site) and TTGCGGCCGCTCAGGTCAGTCAGAATTCCGGCGGCTAGCGATACCGTC, and the hGH polyA using primers CCTGAGCGGCCGCAAAAATCGATtCTGTGCCTTCTAGTTGCCAGC and TGGACCATCCTCTAGACTGCC (underline: *Xba*I site), and cloning these fragments into the two *Xba*I sites of pCMT-SAhygpA-NP21 using In-Fusion cloning reagents (Takara). PGK polyA and PGKpro sequences were amplified from pCMT-SAhygpA-NP21 using the primer set (CTGACCTGAGCGGCCGCAGAAATTGATGATCTATTAAACAATAAAGA and GGTGGACGCGTAGGTCGAAAGGCCCGGAGAT (underline: *Not*I site)). The BSD gene was amplified from pLIB2-pgkBSD [25] using the primer set (ACCTACGCGTCCACCATGCCTTTGTCTCAAGAAGAATC and GAAGGCACAGAATCGATTTAGCCCTCCCACACATAACC (underline: *Cla*I site)). The resulting PGK polyA-PGKpro and BSD fragments were cloned into the *Not*I and *Cla*I sites of pCMT-SA using In-Fusion cloning reagents, generating pCMT-SApA-BSD. pMEEB-FLAG-PIG-P [14] was used for the expression of *PIGP*. pCAGGS-flpE-puro was provided by Dr. Francis Stewart (Dreden University of Technology) [26], and the pBluescript II plasmid was purchased from Agilent.

### Cell Viability Assay

HAP1 cells (5 × 10^3^/well) were cultured for 24 h in 96-well plates, then culture medium was changed to prewarmed medium containing different concentrations of proaerolysin (0, 0.009, 0.019, 0.039, 0.078, 0.156, 0.3125, 0.625, 1.25, 2.5, or 5 nM). After incubation for 3 h at 37°C, viable cells were assessed using the WST-1 cell proliferation assay (Roche) according to the manufacturer’s instructions. Absorption at 450 and 650 nm was measured using a microplate reader (BioRad) to determine the amount of formazan. Percent viability was calculated as:

Viability = 100 × [aerolysin-treated (A450 nm–A650 nm)–background (A450 nm–A650 nm)] / [non-treated (A450 nm–A650 nm)–background (A450 nm–A650 nm)]

### Establishment of Gene-Trapped HAP1 Mutant Cell Population

A gene trap virus was produced by transfecting the Platinum-GP Retroviral Packaging Cell Line in eight 15-cm dishes with a mixture of pCMT-SApA-BSD and pLC-VSVG plasmids. The virus-containing supernatant was concentrated five times using PEG-it virus precipitation solution (System Biosciences) and then mixed with 8 μg/ml of polybrene prior to transfection. HAP1 cells were enriched by the cell sorter FACSAria II (BD Bioscience) and proliferated before mutagenesis. A total of 6 × 10^7^ cells prepared in six-well plates containing 2.5 × 10^6^ cells per well were infected by centrifugation at 2,500 rpm for 2 h at 32°C. Two days after infection, the cells were selected with 6 μg/ml of blasticidin (InvivoGen) for 1 week.

### Enrichment of GPI-negative HAP1 Mutant Population

After selection with blasticidin, mutagenized HAP1 cells (2.4 × 10^8^ cells) were treated with 0.2 nM proaerolysin for 1 day. Surviving cells were cultured, proliferated, and treated again with 0.2 nM proaerolysin for 1 day. Surviving cells were stained with an anti-CD59 antibody followed by PE-conjugated anti-mouse IgG, and CD59-negative cells were enriched by cell sorting using a FACSAria II. The sorted cells were pooled as the GPI-negative cell population (S1 Fig).

### Flow Cytometry

HAP1 cells were harvested, collected, and resuspended in FACS solution (phosphate-buffered saline containing 1% bovine serum albumin and 0.1% NaN_3_). A total of 5 × 10^5^ cells/sample were stained with an anti-CD59 or anti-DAF antibody (10 μg/ml) and PE-conjugated goat anti-mouse IgG. In some cases, cells were stained with FLAER (10^−8^ M). Stained cells were analyzed using the FACSCanto II (BD).

### Electroporation

HAP1 cells (2 × 10^7^ cells) were collected and resuspended in 0.2 ml of OPTI-MEM (Life Technologies). Cells were mixed with 10 μg of plasmids and electroporated once at 250 V for 20 ms using ECM830 (BTX). Alternatively, cells and 10 μg of plasmids were mixed and resuspended in 100 μl of buffer T (Life Technologies) and electroporated at 2000 V for 10 ms twice using Neon (Life Technologies).

### Sequence Analysis of Gene-Trap Insertion Sites in Clonal Cells

Genomic DNA was isolated from 2 × 10^6^ HAP1-GT-C3 cells using the Wizard Genomic DNA purification kit (Promega), then 2 μg was digested with *Hae*III and ligated with the splinkerette adaptor [24], which consists of two oligonucleotides: Spl-top-HaeIII and SplB-BLT-HaeIII (oligonucleotide sequences are listed in S2 Table). DNA fragments were then digested with *Pvu*II, which cleaves the vector sequence between the 3′ long terminal repeat (LTR) and the upstream *Hae*III site, to prevent unwanted vector amplification (S2 Fig). After column purification, the fragments were used as templates for splinkerette polymerase chain reaction (PCR) using the Spl-P1 and LTR-1st primers, followed by nested PCR using the Spl-P2 and LTR-2nd primers (S2 Table). The resulting DNA fragments were separated by agarose gel electrophoresis, cut and extracted from the gel. After phosphorylation of the DNA fragments by T4 polynucleotide kinase (NEB), they were ligated into *Eco*RV sites of pBluescript II and sequenced.

### Sequence Analysis of Gene-Trap Insertion Sites by NGS

Genomic DNA was isolated from 3 × 10^7^ cells using the Wizard Genomic DNA purification kit (Promega), then 15 μg was digested with *Hae*III and ligated with the splinkerette adaptor as before. DNA fragments were digested with *Pvu*II, purified, and used as templates for splinkerette PCR as described above. PrimeSTAR GXL DNA polymerase (Takara) was included in the PCR to reduce amplification bias. Resulting DNA fragments were further amplified by nested PCRs using Spl-P2 and LTR-2nd primers, followed by Rd1Tru-LTR and Rd2Tru-Splink primers, which contain the Illumina sequencing primer sequences. The resulting DNA products contained the end of the 5′ LTR retroviral sequence, followed by the genomic DNA sequence flanking the insertion site ending at the *Hae*III restriction site and part of the splinkerette adaptor sequence. Illumina P5 (AATGATACGGCGACCACCG) and P7 (CAAGCAGAAGACGGCATACGA) adapters and barcode sequences were attached to the products by six cycles of PCR using 10 ng of each of the initial PCR product as template. Single-end sequencing (151-bp) was performed using the HiSeq 2500 system (Illumina). The numbers of reads obtained from non-selected control cells and GPI-negative cells were approximately 7.5 million and 1.7 million, respectively.

### Analysis of Gene-Trap Insertions

FASTQ data files were analyzed using CLC Genomic Workbench software (Qiagen). After quality trimming and removal of the common LTR sequence, all reads were further trimmed from their 3′ ends to a length of 50-bp. These 50-bp reads were mapped onto the human genome (hg19). To exclude ambiguous alignments, all non-specific matched reads were ignored. To eliminate PCR amplification bias and to determine the independent insertion sites, duplicate reads were removed and counted as one read (a unique insertion site). The mapped reads in each gene were further analyzed by the RNA-Seq tool of the CLC Genomics Workbench software. Mismatched reads were excluded to eliminate bias of the mapped reads in each gene caused by PCR or sequencing errors. We mapped 199,043 and 12,183 independent reads to the human genome in non-selected and GPI-negative cell populations, respectively. Of these, 56,839 insertion sites in the non-selected population and 1,681 in the GPI-negative population were found in gene regions. The number of insertions per gene and inactivating insertions (all the forward insertions and reverse insertions in exons) per gene were counted. 34,768 and 1,164 inactivating insertions were identified in non-selected and GPI-negative cell populations, respectively. The amount of enrichment of a particular gene was calculated by comparing the selected with the non-selected population. For each gene, a *P*-value and a *P*-value corrected for the false discovery rate were calculated by the one-sided Fisher’s exact test using R software. A bubble plot was also created using R software.

## Results

### Genetic Screening of GPI Biosynthetic Factors using HAP1 Cells Treated with Proaerolysin

**Fig 1:**
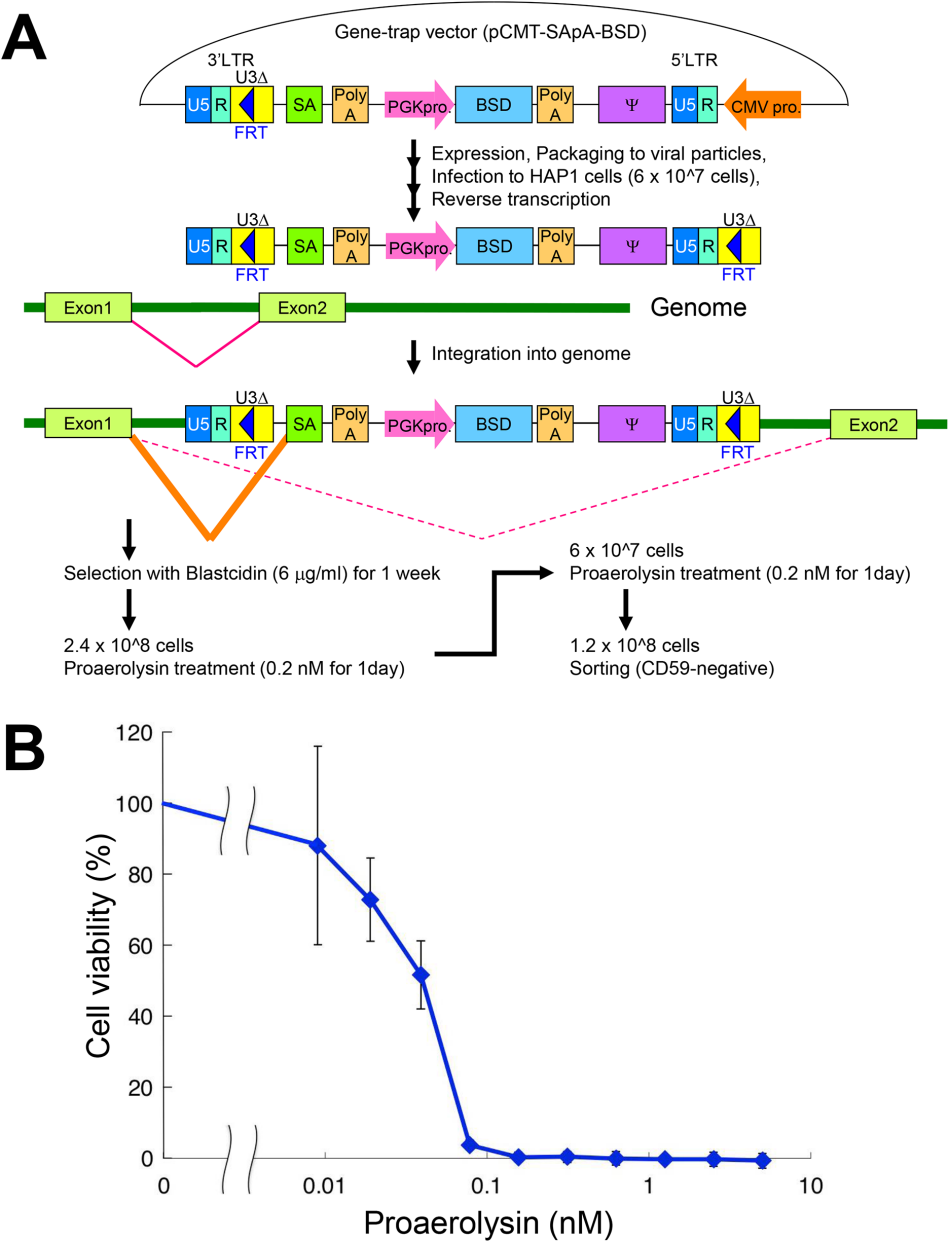
Haploid genetic screening to reveal factors required for GPI biosynthesis using aerolysin sensitivity. A. Structure of the gene trap vector and strategy for enrichment of GPI-negative mutant cells. Retrovirus-based gene trap vectors containing splice acceptor site (SA) and polyadenylation signal (PolyA) were infected into HAP1 cells. The 3′ flippase recognition target (FRT) site of the provirus is copied into the 5′ long terminal repeat (LTR). Mutagenized cells (6 × 10^7^ cells) were selected with 6 μg/ml blasticidin for 1 week. To eliminate the possibility that GPI-negative mutant cells were lost during the screening procedure, the 4 times of infected cells (2.4 × 10^8^ cells) were used for the treatment with 0.2 nM proaerolysin for 1 day. After the first treatment, the survived cells were harvested and cultured in new plates. Then, 6 × 10^7^ cells were treated with 0.2 nM proaerolysin for 1 day again. Resistant cells were proliferated and stained with an anti-CD59 antibody and CD59-negative cells were further enriched by cell sorting. Ψ, packaging signal; PGKpro, PGK promoter; CMV pro, CMV promoter; BSD, blasticidin S-deaminase gene. B. Sensitivity of HAP1 cells to proaerolysin. HAP1 cells were treated with indicated concentrations of proaerolysin (nM) for 3 h. After changing to medium without aerolysin, cell viability was measured by the WST-1 assay and shown in % viability. Viability of cells without proaerolysin treatment was 100%. Data are means ± standard deviation (n = 4).

Recently, Brummelkamp and co-worker reported excellent genetic screening methods using human haploid cells combined with gene trapping [18, 21]. We applied the methods for analysis of genes required for GPI biosynthesis. Retrovirus-based gene trap vector was designed for mutagenesis that confers blasticidin resistance to the cells to ensure vector integration into the genome (Fig 1A). Aerolysin was used to obtain mutant cells defective in the biosynthesis of GPI-APs (Fig 1A). Aerolysin is secreted by the gram-negative bacteria *Aeromonas* and is known to bind to the GPI-anchor itself for cell membrane targeting [26], after which it forms a heptamer and makes a hole in the cell membrane [27]. To determine the cytotoxicity of aerolysin in HAP1 cells, we treated them with various concentrations of proaerolysin (Fig 1B). At concentrations ≥0.15 nM, 99% of cells died, so we used 0.2 nM proaerolysin to obtain cells resistant to aerolysin. We introduced gene trap vectors into the genomes of 6 × 10^7^ HAP1 cells, then treated them twice with proaerolysin to obtain aerolysin-resistant cells (Fig 1A). Since significant amounts of cells still express GPI-APs on the cell surface even after proaerolysin treatment, cells with reduced surface expression of CD59, which is one of the ubiquitously expressed GPI-APs, were obtained (GPI-negative cell pools) using a cell sorter to become the enriched population.

**Fig 2:**
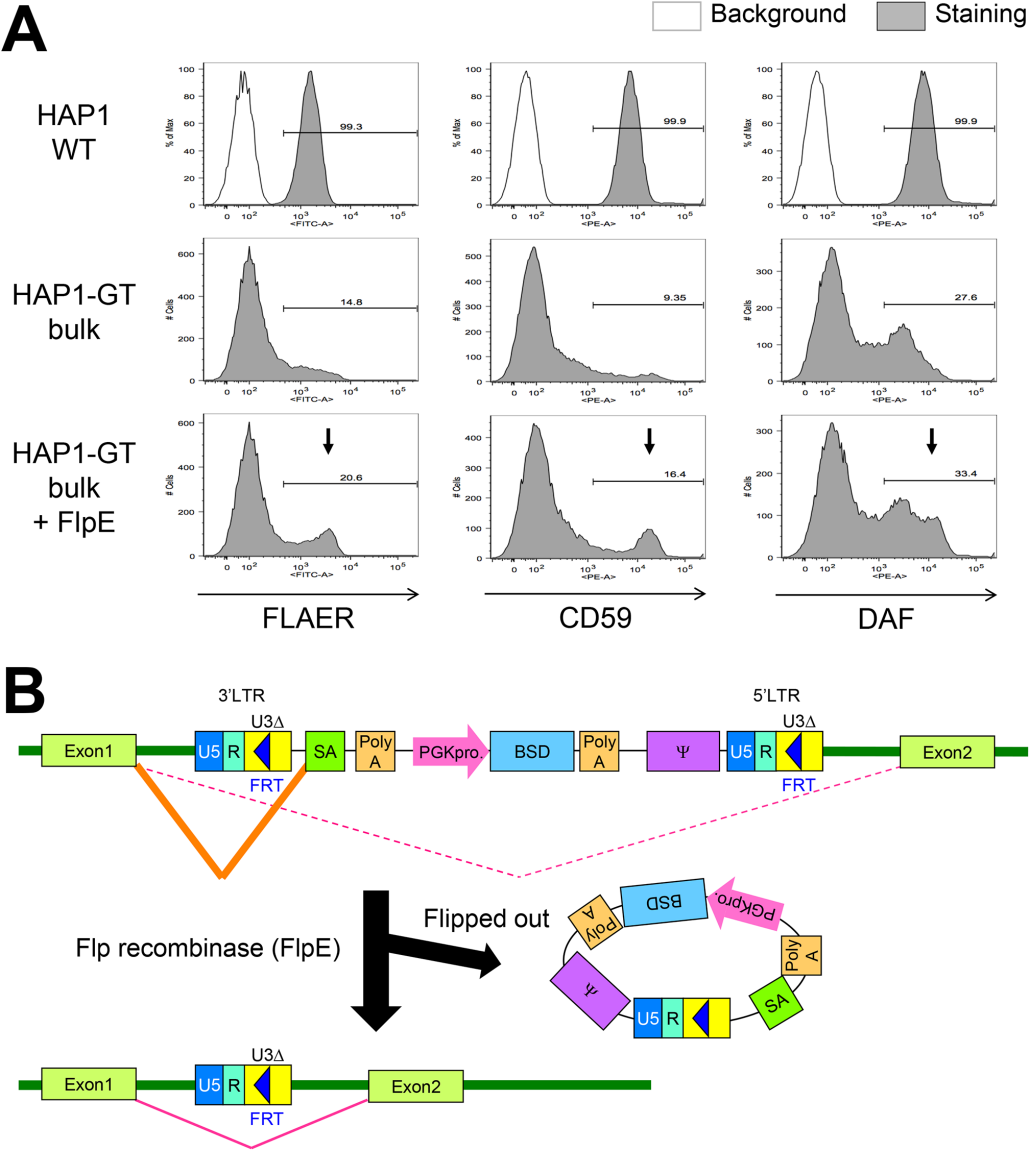
Enrichment and characterization of GPI-negative HAP1 cells. A. After the enrichment of GPI-negative cells, surface GPI-APs in parental HAP1 cells (WT), bulk population of enriched gene-trapped HAP1 cells (HAP1-GT), and the HAP1-GT transfected with Flp recombinase (FlpE) expression plasmid were stained with fluorescent-aerolysin (FLAER), anti-CD59 antibody, or anti-CD55/DAF antibody. A phycoerythrin (PE)-conjugated secondary antibody was used to stain CD59 and DAF. FLAER was detected by flow cytometry using the FITC channel. Arrows indicate cells recovered GPI-APs after FlpE expression. B. Schematic of the elimination of the gene trap vector from the genome by the action of Flp recombinase. Gene splicing and transcription are impaired following insertion of the gene trap vector into the intronic region of a gene in the forward direction. After flipping out of the gene trap vector by Flp recombinase, gene splicing is normalized.

Flow cytometric analysis showed that the surface expression of FLAER and GPI-APs such as CD59 and DAF was greatly reduced in the enriched cells compared with wild-type cells (Fig 2A). If the gene trap insertions had occurred within introns, we would expect that excision of the mutagenic vector components by Flp recombinase using flippase recognition target (FRT) sites in the LTR of the retroviral vector (Fig 2B) would reverse the mutant phenotype. Indeed, some HAP1-GT cells showed restored surface GPI-AP expression following transfection of the Flp recombinase gene (Fig 2A). The observed low restoration rate represented the transfection efficiency and the efficiency of Flp recombinase activity in the excision of insertional sites.

### Characterization and Determination of the Genes Responsible for Clonal GPI-Negative HAP1 Cells

**Fig 3:**
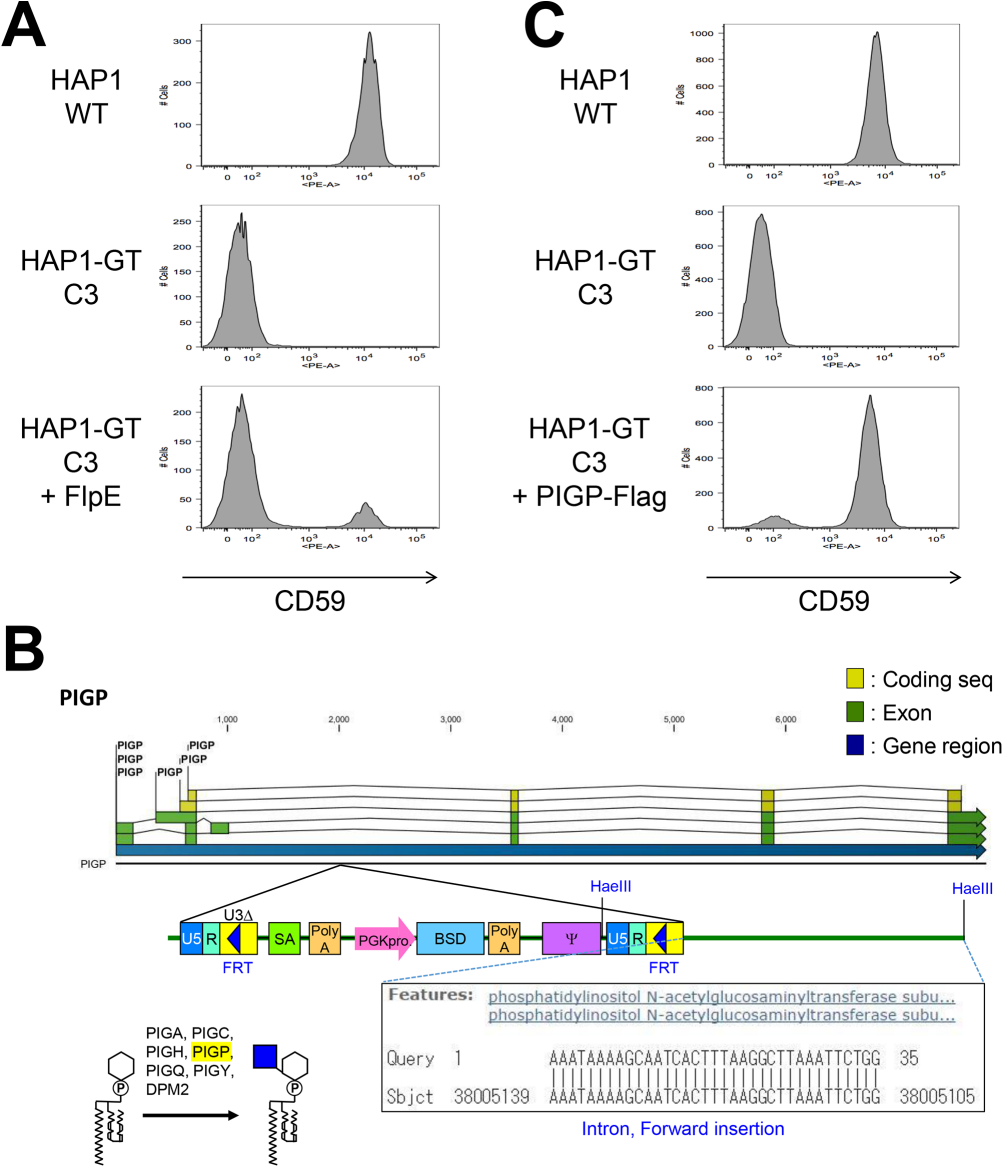
Isolation of a mutant cell line defective in GPI biosynthesis. A. Defective CD59 expression in HAP1-GT clone 3 (C3) and restoration by FlpE expression. Parental HAP1 cells (WT), HAP1-GT-C3 cells, and FlpE-transfected HAP1-GT-C3 were stained with an anti-CD59 antibody, followed by PE-conjugated anti-mouse IgG. B. Determination of the gene-trapped insertion site in HAP1-GT-C3. Schematic of an insertion site in *PIGP*. PIGP protein forms a complex with PIGA, PIGC, PIGH, PIGQ, PIGY, and DPM2 and acts as a GPI-GnT, which is the first reaction for GPI biosynthesis. C. Restoration of CD59 expression in HAP1-GT-C3 by the transfection of *PIGP*. HAP1-GT-C3 cells were electroporated with a PIGP-Flag-expressing plasmid. The surface expression of CD59 was detected in parental HAP1 cells, HAP1-GT-C3, and PIGP-Flag-transfected C3 cells.

We isolated several clonal cell lines from the GPI-negative cell population, including HAP1-GT clone 3 (C3) which showed no surface expression of GPI-APs (Fig 3A). GPI-AP expression could, however, be rescued by transfection of the Flp recombinase gene. We identified six insertion sites of the gene trap vector in the genome of HAP1-GT-C3 cells (S3 Fig). Three of these were found in intergenic regions, one matched a bacterial artificial chromosome sequence derived from chromosome 3, one was inserted in the reverse direction of an intron so would not affect splicing, and the last was integrated in the forward direction in an intronic region of *PIGP* (Fig 3B), which encodes a subunit of GPI-GnT. Transfection of a complete *PIGP* gene restored the surface expression of GPI-APs (Fig 3C), showing that the screening was accurate.

### Comprehensive Analysis of Genes Required for GPI Biosynthesis

**Fig 4:**
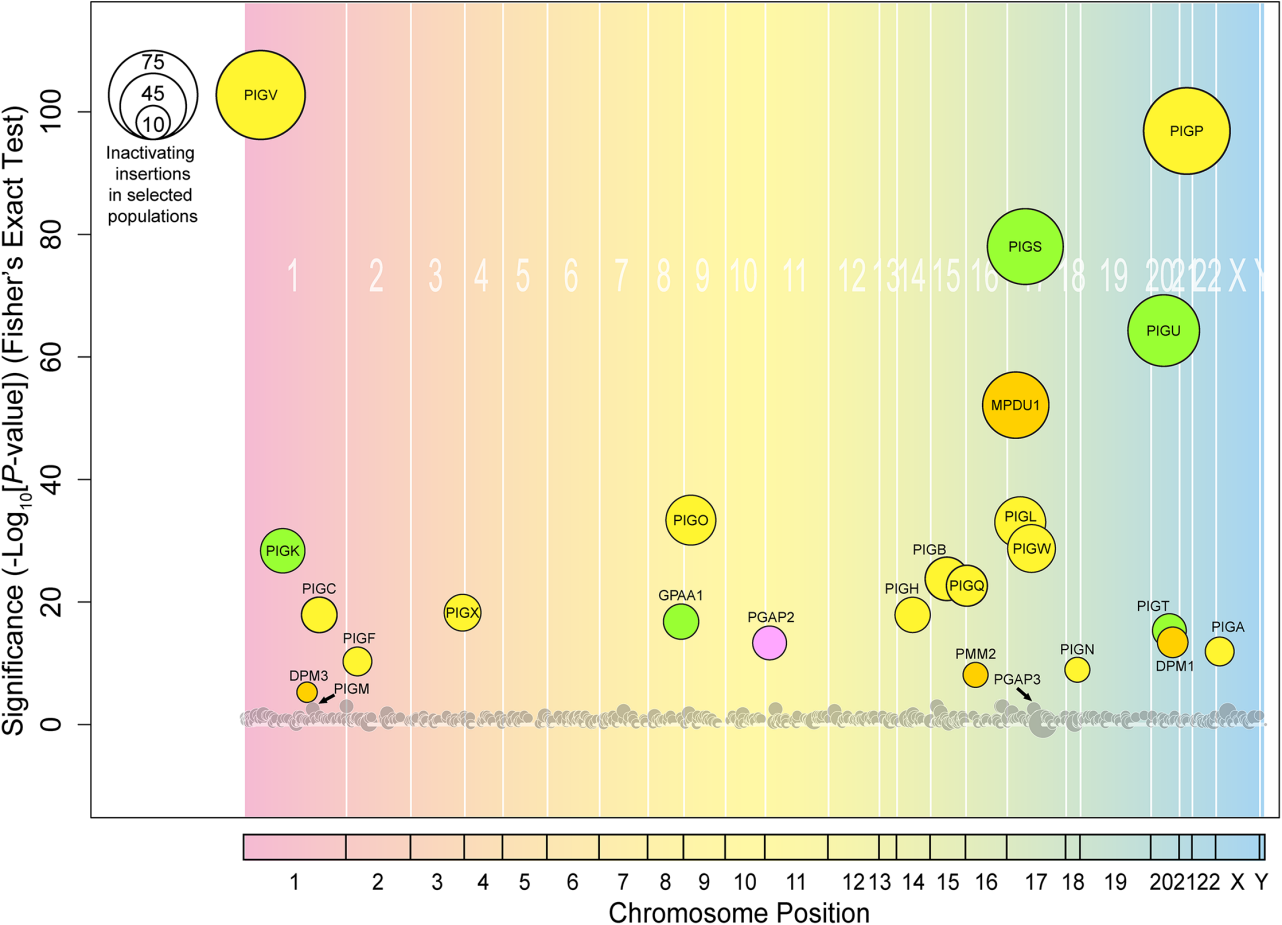
Haploid genetic screening for factors required for GPI biosynthesis. The significance of the enrichment of gene trap insertions in enriched GPI-negative cells compared with the non-selected population was plotted as a bubble plot. The horizontal line shows the chromosomal position of the genes, and the vertical line the significance of enrichment of each gene (*P*-value). The size of the bubble shows the number of inactivating insertion sites in enriched GPI-negative populations. Genes significantly enriched in the GPI-negative population (*P*<0.001) are colored. Yellow bubbles indicate genes encoding the biosynthesis of GPI intermediates in the ER, green bubbles the genes required for GPI-transamidation, orange bubbles the genes involved in Dol-P-Man synthesis and utilization, and pink bubbles the gene involved in GPI-AP remodeling. The bubbles of PIGM and PGAP3 (arrows) were close to the significance limit.

We next tried to comprehensively identify insertion sites of the gene trap vector in GPI-negative cell pools. Genomic DNA was extracted from cell populations without selection or GPI-negative cell populations, and the Illumina HiSeq was used to determine the insertion sites (S1 and S2 Figs). The enrichment of insertion sites between GPI-negative and non-selected populations was analyzed, enabling us to rearrange genes according to their significance. We found that the top 23 genes enriched in the GPI-negative population were known to be directly or indirectly involved in GPI biosynthesis (Fig 4 and S3 Table). Most were phosphatidylinositol glycan genes (*PIG*) [27], although post-GPI-attachment to protein gene 2 (*PGAP2*), *DPM1*, *DPM3*, *MPDU1*, and *PMM2* were also enriched. *PIGM* and *PGAP3* were enriched to a lesser extent, although this was not significant (S3 Table).

**Fig 5:**
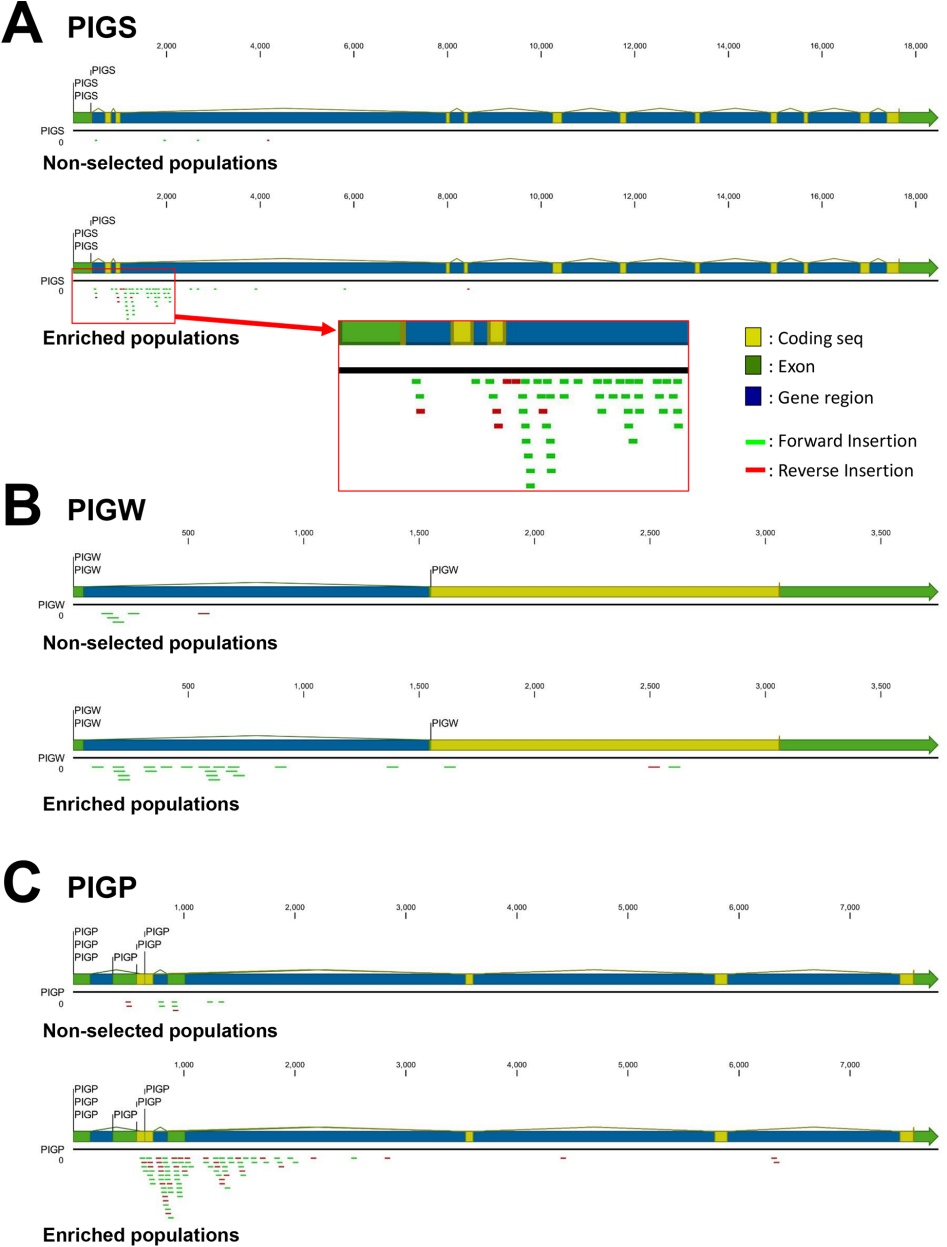
Insertion sites in GPI biosynthetic gene loci. Insertion sites of PIGS (A), PIGW (B) and PIGP (C) gene regions in the non-selected and enriched GPI-negative populations. The schematic was constructed using CLC genomics workbench software. Insertions in 5’-region of PIGS gene are shown in enlarged red square. Forward insertions and reverse insertions are shown in green and red, respectively.

We undertook further analysis of a number of genes. To count the insertion sites in each genes, CLC Genomics Workbench software was used as described in “Materials and methods” part. In *PIGS*, four independent insertion sites were identified in the non-selected population, of which three were inactivating (Fig 5A). In the GPI-negative population, a total of 58 insertion sites were identified, of which 55 were inactivating. In the case of *PIGW* in the non-selected population, five independent insertion sites were identified, of which four were inactivating; this compares with 22 in the GPI-negative population, all of which were inactivating (Fig 5B). In *PIGP*, nine independent insertion sites were identified in the non-selected population, all of which were inactivating (Fig 5C). In the GPI-negative population, a total of 92 insertion sites were identified, of which 72 were inactivating.

## Discussion

**Fig 6:**
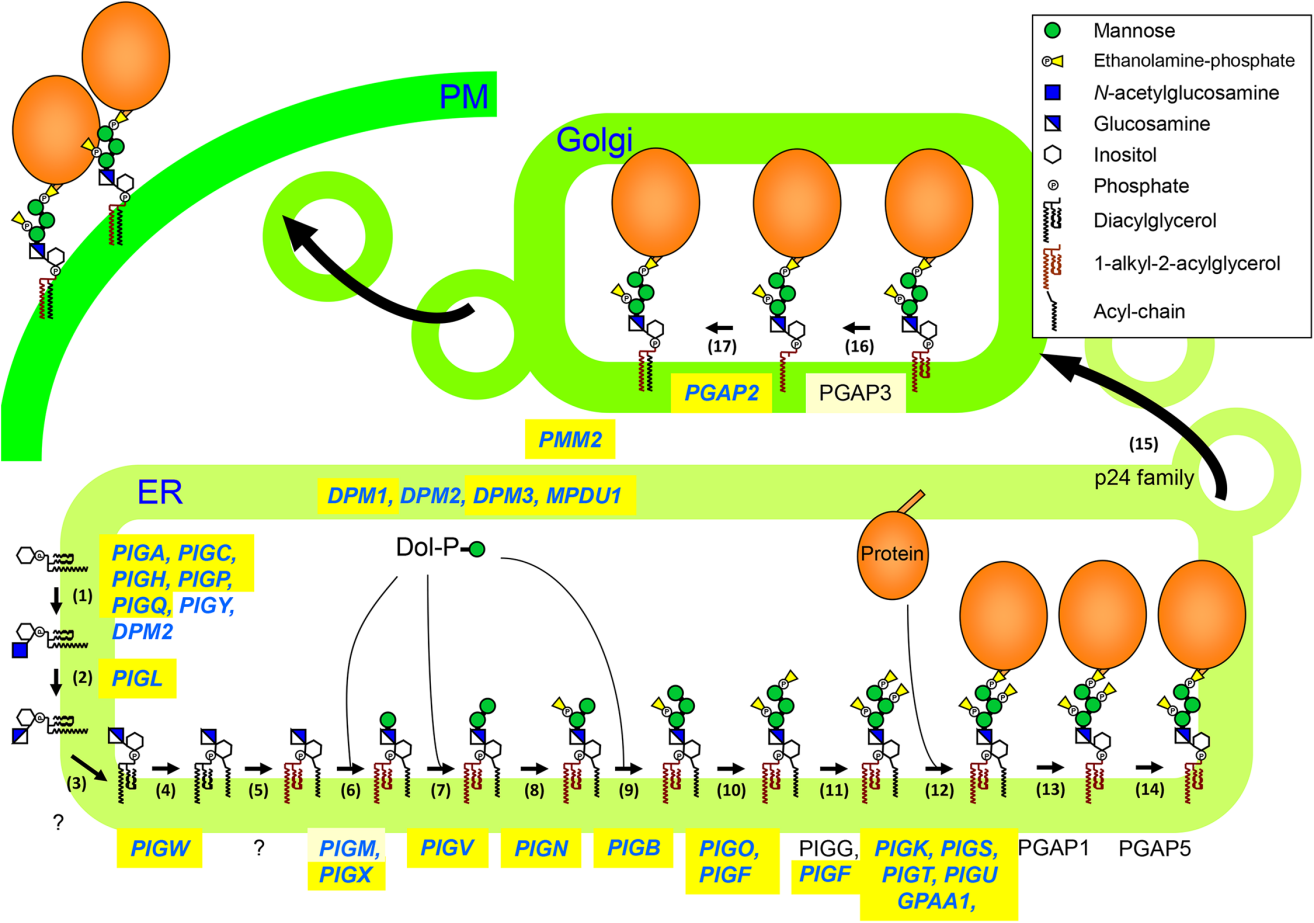
Biosynthesis, remodeling, and transport of GPI-APs in mammalian cells. GPI is biosynthesized in the ER from PI by the stepwise addition of sugars, an acyl-chain and phospho-ethanolamines. After GPI is transferred to a protein, GPI moieties are remodeled during transport. Genes colored in blue (*italic*) have been shown to decrease surface expression of GPI-APs in mutant cells. Genes significantly enriched and genes weakly enriched in this study are highlighted in yellow and light yellow, respectively.

In this study, we performed a genome-wide screen for GPI biosynthesis using human haploid cells and gene trap vectors. Treatment with proaerolysin enabled mutants defective in the GPI biosynthetic pathway to be enriched. At least 30 genes were shown to be involved in the biosynthesis of GPI-APs, either directly or indirectly (Fig 6 and S1 Table). Of these, 26 genes demonstrated decreased surface expression of GPI-APs when they were disrupted (Fig 6, shown in blue). Screening identified 23 genes required for GPI biosynthesis that were significantly enriched in the GPI-negative cell population. These included *PGAP2*, which is required for GPI fatty acid remodeling in the Golgi. In *PGAP2* mutant CHO cells, the surface expression of GPI-APs was significantly decreased because lyso-forms of GPI-APs (the product in step 16 shown in Fig 6) are transported and released from the plasma membrane into the medium soon after arrival [28]. Other enriched genes were *DPM1*, *DPM3*, *MPDU1*, and *PMM2*, while *PGAP3* and *PIGM* were weakly enriched. DPM1 and DPM3 are required for the synthesis of Dol-P-Man, which is used as a substrate in the mannosylation of GPI precursors in the ER, while MPDU1 functions in the utilization of Dol-P-Man [29]. Mutations in *DPM1*, *DPM3*, and *MPDU1* were previously reported to impair the surface expression of GPI-APs [30–32]. *PMM2* encodes phosphomannomutase 2, which catalyzes the isomerization of mannose-6-phosphate to mannose-1-phosphate. This is subsequently converted to GDP-mannose, which is utilized for mannosylation and Dol-P-Man synthesis. Mutations in *PMM2* cause the most common congenital disorder of glycosylation, known as CDG-Ia or PMM2-CDG, although the partial loss of PMM2 activity has little effect on the expression of GPI-APs in patient cells [33]. *PIGM* is a small gene (4322 bp) with no introns that encodes a protein required for the transfer of the first Man to the GPI intermediate together with PIGX. PGAP3 is required for the fatty acid remodeling of GPI-APs and is critical for the nano-clustering of GPI-APs on the cell membrane [34–36]. In *Pgap3* ^-/-^ mouse embryonic fibroblasts, proaerolysin cytotoxicity is reduced, probably because of slow oligomerization of aerolysin on the cell membrane due to low clustering of GPI-APs containing an unsaturated fatty acid [37].

Two genes, *DPM2* and *PIGY*, were not enriched in our screening, although their mutants show decreased expression of GPI-APs on the cell surface [32, 38]. We detected one inactivating insertion site in *PIGY* gene of the GPI-negative population, but not non-selected population (S3 Table). It is conceivable that because they are relatively small (2821 bp for *PIGY* and 3390 bp for *DPM2*), gene trap vectors might not readily integrate into these genes. Alternatively, insertion of retroviral vectors may have preferences to specific genome regions as previously reported [39]. Similar findings were also observed in the screening of haploid embryonic stem cells mutagenized by N-ethyl-N-nitrosourea, so it was concluded that the mutation rate for each gene is dependent on the length of the coding sequence [40]. More recently, a haploid genetic screen for the GPI biosynthetic pathway similar to ours has been reported in which CD59-negative or Prion protein-negative cells were enriched from gene-trapped HAP1 cells [41]. In the latter study, three additional genes were found. *SEC62* and *SEC63*, which encode subunits of the ER protein translocon complex, were enriched in the Prion protein-negative cell population and *SPPL3* was enriched in the CD59-negative cell population. It has been reported that Prion proteins and GPI-APs in yeast require the SEC62 and SEC63-dependent translocation system to enter into the ER lumen [42–44]. Because aerolysin binds to GPI-anchor itself for cell targeting, factors generally required for GPI-APs would be enriched in our screening, however those required for expression of specific GPI-APs may not be enriched. In addition, it has been reported that the GPI-anchor on Prion proteins does not seem to be recognized by aerolysin [45]. Actually, the factors such as *SEC62* and *SEC63* were not enriched in our screening. Conversely, *PGAP3* was found only in our screening consistent with a previous report that *PGAP3* deletion caused mild resistance to proaerolysin [37].

Details of the factors required in the GPI biosynthetic pathway have not been fully clarified (S1 Table). For example, glucosamine-PI is flipped into the ER luminal side from the ER cytoplasmic face in the early stages of the pathway, but the putative GPI-flippase has not yet been identified [2]. Our study and those of others failed to show the enrichment of any suitable candidate genes [40, 41, 46]. There could be several reasons for this. First, it is possible that the GPI-flippase gene is essential for cell growth, although no other gene involved in GPI biosynthesis is essential for growth at the cellular level. Second, the gene required for flipping might be dispensable for the surface expression of GPI-APs, so even if mutated, GPI-APs surface expression would be normal. Third, the flippase gene could be duplicated in the genome, so a mutation in one gene would be compensated for by the other. Fourth, if the gene encoding flippase is very short, insertions would not be enriched during screening, as seen for *DPM2* and *PIGY*. Therefore, to identify all regulatory factors involved in the GPI biosynthetic pathway, different strategies or new assay systems are essential.

One of the difficulties of forward genetics in mammalian cells is the ploidy of the chromosomes, which is at least diploid in mammalian cell lines. Therefore, even if one allele is mutated, the corresponding allele on the homologous chromosome remains intact, so the phenotype is unaffected. This multi-ploidy makes it difficult to conduct genetic analysis. In mammalian cells, forward genetic approaches using RNA interference have been widely used [47, 48]. However, genes encoding enzymes can be difficult to isolate using this technique because only a small amount of enzyme is sufficient for the reaction to occur. Moreover, substantial off-targeting effects can be observed [49, 50]. Recently, CRISPR/Cas9 systems have been used to overcome this [46, 51–53]. Although the screening achieved with these systems appears to be efficient, not all genes can be disrupted and one also need to care about the off targeting effects.

Genetic screening using HAP1 cells combined with gene trapping can therefore be used as a high throughput and reliable alternative to yeast genetics [18, 21, 54]. Although gene trap-independent mutations or genetic revertants are often observed during the screening process, they can be eliminated by removing the SA site in the trapped region from the genome by expressing Flp recombinase. This is also useful to confirm whether the mutant cells that are isolated or enriched show the phenotypes of interest.

## Supporting Information

S1 Fig Strategy for screening to identify factors required for GPI biosynthesis. Mutagenized HAP1 cells were enriched by aerolysin resistance. Genomic DNA was purified from both non-selected and GPI-negative enriched cell populations. After the amplification of vector insertion sites, DNA fragments were sequenced and analyzed. Independent reads in each gene were counted and compared between non-selected and GPI-negative enriched populations.
(TIF)

S2 Fig Strategy for determining vector insertion sites. Genomic DNA was digested with *Hae*III, then ligated to the splinkerette adaptor. Ligated DNA fragments were digested with *Pvu*II to cleave the vector sequence between the 3′ LTR and the upstream *Hae*III site. Fragments were amplified by PCR, then ligated into the *Eco*RV site of pBluescript II, and sequenced to determine insertion sites of clonal cells. For analysis of gene trap insertion sites by NGS, the adaptor and barcode sequence were attached to the fragments and sequenced.
(TIF)

S3 Fig Insertion sites of gene trap vector in HAP1-GT-C3. DNA fragments amplified from the HAP1-GT-C3 genome were phosphorylated and ligated into the *Eco*RV site of pBluescript II. The resulting plasmid was sequenced and the insert sequences underwent a BLAST search on the NCBI website. A total of six different sequences matching the human genome database were identified (see also Fig 3B).
(TIF)

S1 Table GPI biosynthetic genes. (PDF)

S2 Table Oligonucleotides for preparation of DNA fragments containing gene-trap insertion sites. (PDF)

S3 Table Result of haploid screen for GPI biosynthesis. (XLS)
