# Supplementary figures and images for: Genome-Wide Screening of Genes Required for Glycosylphosphatidylinositol Biosynthesis
Source: PLoS One. 2015 Sep 18;10(9):e0138553. doi: 10.1371/journal.pone.0138553 (PMC4575048; doi:10.1371/journal.pone.0138553)

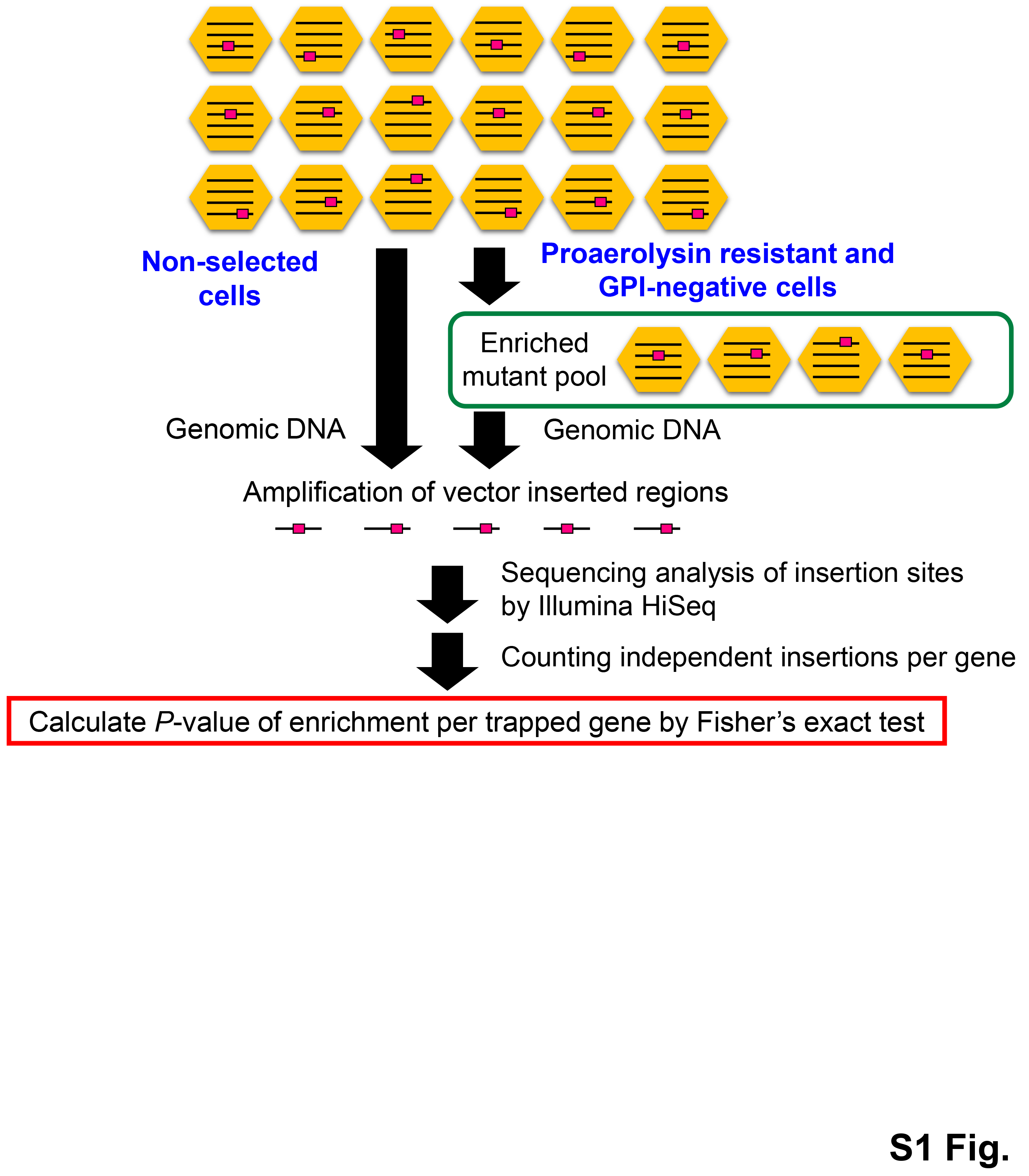

Supplement: S1 Fig — Mutagenized HAP1 cells were enriched by aerolysin resistance. Genomic DNA was purified from both non-selected and GPI-negative enriched cell populations. After the amplification of vector insertion sites, DNA fragments were sequenced and analyzed. Independent reads in each gene were counted and compared between non-selected and GPI-negative enriched populations. (TIF) [file pone.0138553.s001.tif]

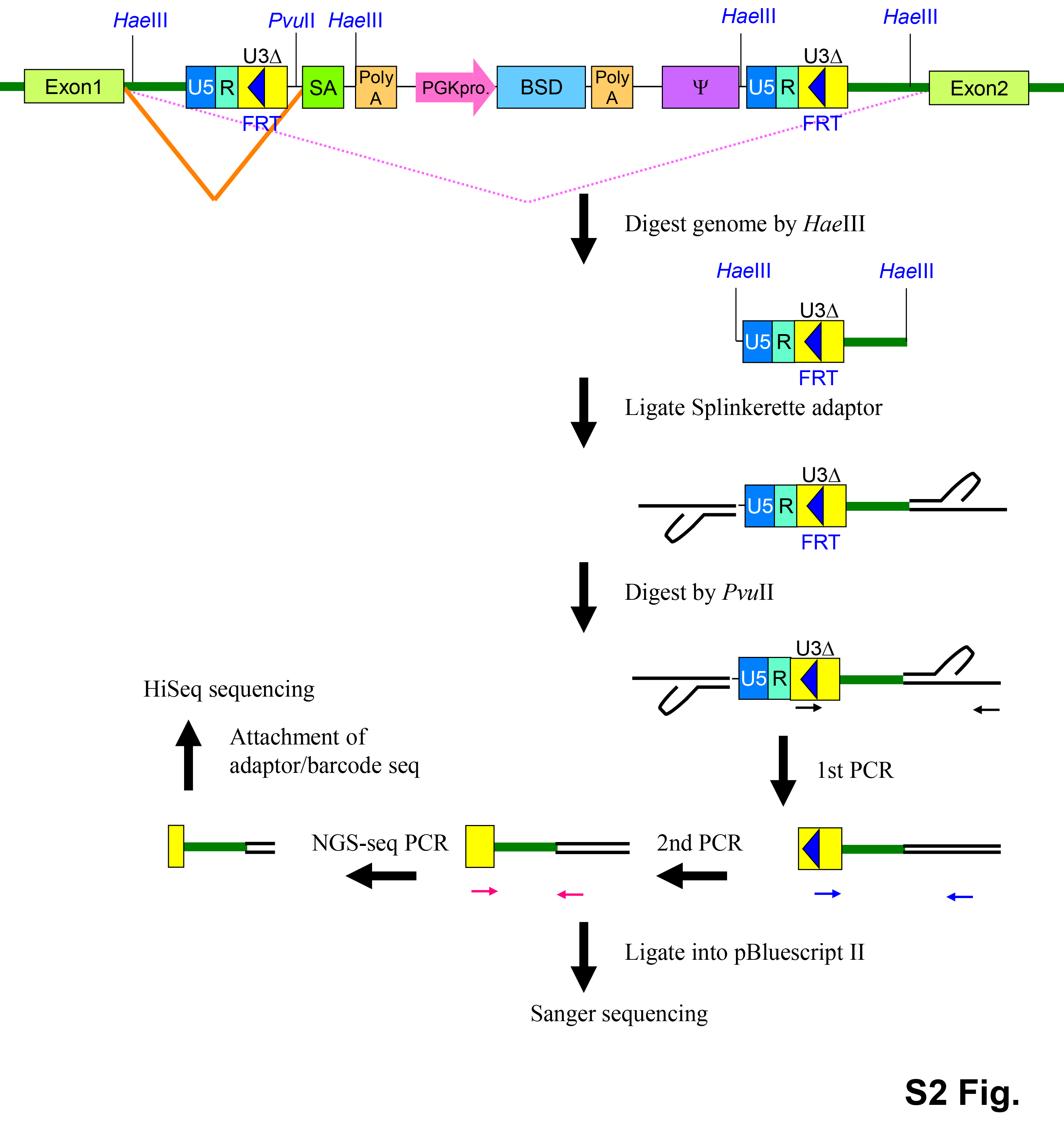

Supplement: S2 Fig — Genomic DNA was digested with HaeIII, then ligated to the splinkerette adaptor. Ligated DNA fragments were digested with PvuII to cleave the vector sequence between the 3′ LTR and the upstream HaeIII site. Fragments were amplified by PCR, then ligated into the EcoRV site of pBluescript II, and sequenced to determine insertion sites of clonal cells. For analysis of gene trap insertion sites by NGS, the adaptor and barcode sequence were attached to the fragments and sequenced. (TIF) [file pone.0138553.s002.tif]

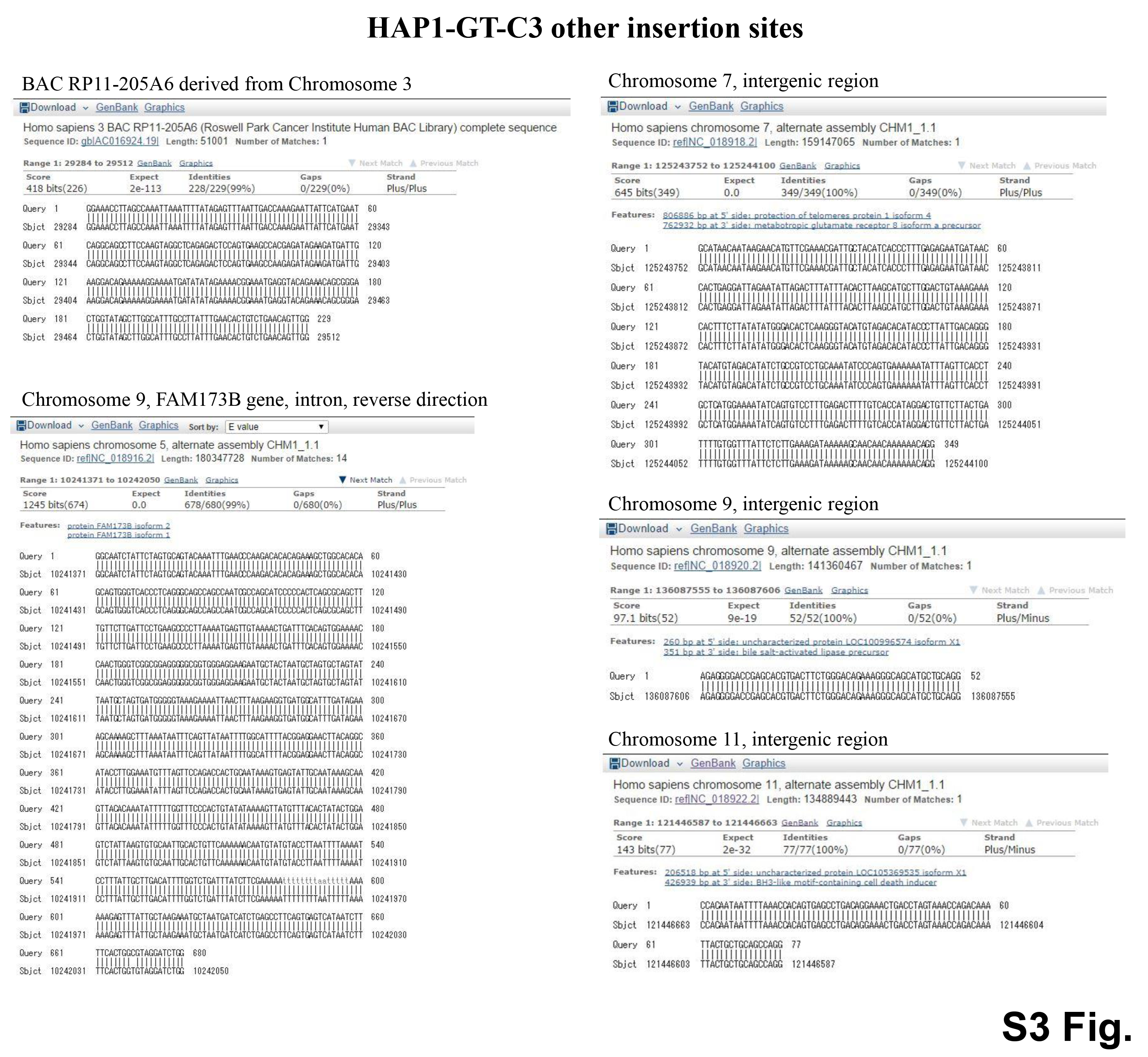

Supplement: S3 Fig — DNA fragments amplified from the HAP1-GT-C3 genome were phosphorylated and ligated into the EcoRV site of pBluescript II. The resulting plasmid was sequenced and the insert sequences underwent a BLAST search on the NCBI website. A total of six different sequences matching the human genome database were identified (see also Fig 3B). (TIF) [file pone.0138553.s003.tif]
