# Supplementary material for: Genome-Wide Screening of Genes Required for Glycosylphosphatidylinositol Biosynthesis
Source: PLoS One. 2015 Sep 18;10(9):e0138553. doi: 10.1371/journal.pone.0138553 (PMC4575048; doi:10.1371/journal.pone.0138553)
Supplement: S1 Table — (PDF) [file pone.0138553.s004.pdf]

Table S1: GPI biosynthetic genes

Step	Enzyme	Donor	Human gene	Year	Locus	Deficiency	*S. cerevisiae* gene	Year	First characterization	Reference (yeast)	Reference (mammals)
1	GPI-GlcNAc transferase (GPI-GnT)	UDP-GlcNAc	*PIGA*	1993	Xp22.2	PNH、MCAHS	*GPI3*	1995	Mammalian genetics	http://www.ncbi.nlm.nih.gov/pubmed/7768896	http://www.ncbi.nlm.nih.gov/pubmed/7680492
			PIGC	1996	1q23-q25		*GPI2*	1995	Yeast genetics	http://www.ncbi.nlm.nih.gov/pubmed/7768896	http://www.ncbi.nlm.nih.gov/pubmed/8806613
			PIGH	1993	14q24.1		*GPI15*	2001	Mammalian genetics	http://www.ncbi.nlm.nih.gov/pubmed/11746600	http://www.ncbi.nlm.nih.gov/pubmed/8407896
			PIGP	2000	21q22.2		*GPI19*	2005	Biochemical analysis	http://www.ncbi.nlm.nih.gov/pubmed/16278447	http://www.ncbi.nlm.nih.gov/pubmed/10944123
			PIGQ	1996	16p13.3	Ohtahara Syndrome	*GPI1*	1994	Yeast genetics	http://www.ncbi.nlm.nih.gov/pubmed/8144596	http://www.ncbi.nlm.nih.gov/pubmed/8806613
			PIGY	2005	4q22.1		*ERI1*	2004	Yeast genetics	http://www.ncbi.nlm.nih.gov/pubmed/15163411	http://www.ncbi.nlm.nih.gov/pubmed/16162815
			DPM2	2000	9q34.13	DPM2-CDG	-		Biochemical analysis*		http://www.ncbi.nlm.nih.gov/pubmed/10944123
2	GlcNAc-PI deacetylase		*PIGL*	1997	17p12-p11.2	CHIME syndrome	*GPI12*	1999	Mammalian genetics	http://www.ncbi.nlm.nih.gov/pubmed/10085243	http://www.ncbi.nlm.nih.gov/pubmed/9188481
3	Flippase ?		Not identified						Not identified		
4	Ino acyltransferase	Palmitoyl-CoA	*PIGW*	2003	17q12	West Syndrome	*GWT1*	2003	Mammalian genetics	http://www.ncbi.nlm.nih.gov/pubmed/12714589	http://www.ncbi.nlm.nih.gov/pubmed/14517336
5	PI alkyl-acyl exchange enzyme	?	Not identified				-	-	Not identified		
6	α1,4-Man transferase I (GPI-MT I)	Dol-P-Man	*PIGM*	2001	1q23.1	Inherited GPI deficiency	*GPI14*	2005	Mammalian genetics	http://www.ncbi.nlm.nih.gov/pubmed/16134120	http://www.ncbi.nlm.nih.gov/pubmed/11226175
			PIGX	2005	3q29		*PBN1*	2005	Mammalian genetics	http://www.ncbi.nlm.nih.gov/pubmed/15635094	http://www.ncbi.nlm.nih.gov/pubmed/15635094
							ARV1				
7	α1,6-Man transferase II (GPI-MT II)	Dol-P-Man	*PIGV*	2005	1p36.11	HPMR	*GPI18*	2005	Mammalian genetics	http://www.ncbi.nlm.nih.gov/pubmed/15623507	http://www.ncbi.nlm.nih.gov/pubmed/15623507
			-				*PGA1*				
8	EtNP transferase I (GPI-ET I)	PE	*PIGN*	1999	18q21.33	MCAHS	*MCD4*	1999	Yeast genetics	http://www.ncbi.nlm.nih.gov/pubmed/10069808	http://www.ncbi.nlm.nih.gov/pubmed/10574991
9	α1,2-Man transferase III (GPI-MT III)	Dol-P-Man	*PIGB*	1996	15q21.3		*GPI10*	1998	Mammalian genetics	http://www.ncbi.nlm.nih.gov/pubmed/9576863	http://www.ncbi.nlm.nih.gov/pubmed/8861954
M4	α1,2-Man transferase IV (GPI-MT IV)	Dol-P-Man	*PIGZ* (*SMP3*)	2004	3q29		*SMP3*	2001	Yeast genetics	http://www.ncbi.nlm.nih.gov/pubmed/11356840	http://www.ncbi.nlm.nih.gov/pubmed/15208306
10	EtNP transferase III (GPI-ET III)	PE	*PIGO*	2000	9p13.3	HPMR	*GPI13*	2000	Yeast genetics	http://www.ncbi.nlm.nih.gov/pubmed/10823837	http://www.ncbi.nlm.nih.gov/pubmed/10781593
			PIGF		2p16-p21		*GPI11*		Mammalian genetics		
11	EtNP transferase II (GPI-ET II)	PE	*PIGG* (*GPI7*)	2005	4p16.3		*GPI7*	1999	Yeast genetics	http://www.ncbi.nlm.nih.gov/pubmed/10329735	http://www.ncbi.nlm.nih.gov/pubmed/10329735
			PIGF	1993	2p21-p16		*GPI11*	2000	Mammalian genetics	http://www.ncbi.nlm.nih.gov/pubmed/10793139	http://www.ncbi.nlm.nih.gov/pubmed/8463218
12	GPI transamidase (GPI-TA)		*PIGK*	2000	1p31.1		*GPI8*	1996	Yeast genetics	http://www.ncbi.nlm.nih.gov/pubmed/8978684	http://www.ncbi.nlm.nih.gov/pubmed/10793132
			GPAA1	2000	8q24.3		*GAA1*	1995	Yeast genetics	http://www.ncbi.nlm.nih.gov/pubmed/7730400	http://www.ncbi.nlm.nih.gov/pubmed/10793132
			PIGS	2001	17p13.2		*GPI17*	2001	Biochemical analysis	http://www.ncbi.nlm.nih.gov/pubmed/11483512	http://www.ncbi.nlm.nih.gov/pubmed/11483512
			PIGT	2001	20q12-q13.2	Inherited GPI deficiency, PNH	*GPI16*	2001	Biochemical analysis	http://www.ncbi.nlm.nih.gov/pubmed/11483512	http://www.ncbi.nlm.nih.gov/pubmed/11483512
			PIGU	2003	20q11.22		*GAB1*	2003	Mammalian genetics	http://www.ncbi.nlm.nih.gov/pubmed/12802054	http://www.ncbi.nlm.nih.gov/pubmed/12802054
13	Ino deacylase		*PGAP1*	2004	2q33.1	Inherited GPI deficiency	*BST1*	2004	Mammalian genetics	http://www.ncbi.nlm.nih.gov/pubmed/14734546	http://www.ncbi.nlm.nih.gov/pubmed/14734546
14	EtNP phosphodiesterase		*PGAP5* (*MPPE1*)	2009	18p11.21		*TED1*	2015	Mammalian genetics	http://www.ncbi.nlm.nih.gov/pubmed/25557665	http://www.ncbi.nlm.nih.gov/pubmed/19837036
							CDC1	2014		http://www.ncbi.nlm.nih.gov/pubmed/25165136	
16	GPI phospholipase A2		*PGAP3*	2007	17q12	HPMR	*PER1*	2006	Mammalian genetics	http://www.ncbi.nlm.nih.gov/pubmed/17021251	http://www.ncbi.nlm.nih.gov/pubmed/17314402
17	Lyso-GPI acyltransferase	Stearyl-CoA ?	*PGAP2*	2006	11p15.5	HPMR	*CWH43-N ?*		Mammalian genetics		http://www.ncbi.nlm.nih.gov/pubmed/16407401
			Not identified				*GUP1*	2006		http://www.ncbi.nlm.nih.gov/pubmed/16597698	
CR	Ceramide remodelase	Ceramide ?	*CWH43-C ?*		4p11		*CWH43*	2007		http://www.ncbi.nlm.nih.gov/pubmed/17761529	
	Dol-P-Man (DPM) synthase		*DPM1*	1998	20q13.13	DPM1-CDG/CDG-Ie	*DPM1*	1988	Yeast genetics	http://www.ncbi.nlm.nih.gov/pubmed/3053713	http://www.ncbi.nlm.nih.gov/pubmed/9535917
			DPM2	1998	9q34.13	DPM2-CDG	-		Mammalian genetics*		http://www.ncbi.nlm.nih.gov/pubmed/9724629
			DPM3	2000	1q22	DPM3-CDG/CDG-Io	-		Biochemical analysis		http://www.ncbi.nlm.nih.gov/pubmed/10835346
	DPM/Dol-P-Glucose utilization		*MPDU1*	1996	17p13.1-p12	MPDU1-CDG/CDG-If	-		Mammalian genetics		http://www.ncbi.nlm.nih.gov/pubmed/8663248
	Phosphomannomutase 2		*PMM2*	1997	16p13	PMM2-CDG/CDG-Ia	*SEC53*	1988	Yeast genetics	http://www.ncbi.nlm.nih.gov/pubmed/3288631	http://www.ncbi.nlm.nih.gov/pubmed/9140401

Each steps is corresponded to Figure 6

PNH: paroxysmal nocturnal hemoglobinuria,

HPMR: hyperphosphatasia with mental retardation syndrome,

CHIME: coloboma, congenital heart disease, ichthyosiform dermatosis, mental retardation, and ear anomalies syndrome,

MCAHS: multiple congenital anomalies-hypotonia-seizures syndrome,

CDG: Congenital disorder of glycosylation.

: Genes significantly enriched in this study

: Genes not significantly enriched, but listed up higher ranking

* DPM2 was first identified as a component of DPM synthase by mammalian genetics and later found as a component of GPI-GnT by biochemical analysis of the enzyme complex.
