# Supplementary material for: Genome-Wide Screening of Genes Required for Glycosylphosphatidylinositol Biosynthesis
Source: PLoS One. 2015 Sep 18;10(9):e0138553. doi: 10.1371/journal.pone.0138553 (PMC4575048; doi:10.1371/journal.pone.0138553)
Supplement: S2 Table — (PDF) [file pone.0138553.s005.pdf]

Table S2. Oligonucleotides for preparation of DNA fragments containing gene-trap insertion sites

Usage	Primer name	Sequence
**Splinkerette Adaptor**	Spl-top1-HaeIII	CGAATCGTAACCGTTCGTACGAGAATTCGTACGAGAATCGCTGTCCTCTCCAACGAGCCAAGG
	SplB-BLT1-HaeIII	CCTTGGCTCGTTTTTTTTTGCAAAAA
**1st nested PCR**	LTR-1st	AGTGTATGTAAACTTCTGACCCACTGG
	Spl-P1	CGAATCGTAACCGTTCGTACGAGAA
**2nd nested PCR**	LTR-2nd	CTTGTGTCATGCACAAAGTAGATGTCC
	Spl-P2	TCGTACGAGAATCGCTGTCCTCTCC
**NGS-seq PCR**	Rd1Tru-LTR	ACACTCTTTCCCTACACGACGCTCTTCCGATCTGCTAGCTTGCCAAACCTACAGGTGGG
	Rd2Tru-Splink	GTGACTGGAGTTCAGACGTGTGCTCTTCCGATCTGCTGTCCTCTCCAACGAGCCAAGG

#Red underlines are illumina sequence primers
